# The impact of social media addiction on recreation anxiety in individuals

**DOI:** 10.3389/fpsyg.2026.1860004

**Published:** 2026-07-10

**Authors:** Ramazan Özavci

**Affiliations:** Department of Recreation, Faculty of Sport Sciences, Bingol Universitesi, Bingöl, Türkiye

**Keywords:** digital wellbeing, leisure behavior, recreation, social media, social media addiction, structural equation modeling

## Abstract

**Aim:**

The aim of this study is to investigate the impact of social media addiction on recreation anxiety in individuals.

**Methods:**

This research was conducted using the descriptive survey model, one of the quantitative research methods. The study was carried out with 399 volunteer adult participants (160 males and 239 females) living in Çanakkale, selected through the convenience sampling method based on voluntary participation. While descriptive information was collected via a personal information form, social media addiction data were obtained using the 7-item, single-dimensional ‘social media addiction’ scale. Recreation anxiety data were gathered using the ‘recreation anxiety’ measurement tool, consisting of 25 items and 6 sub-dimensions. After conducting normality, validity, and reliability analyses of the data, the established theoretical model and hypotheses were tested using the Structural Equation Modeling (SEM) method.

**Results:**

As a result of the Structural Equation Modeling, all hypotheses were found to be significant (*p* < 0.001). The findings indicate that the strongest impact of social media addiction is on “responsibility anxiety,” a sub-dimension of the recreation anxiety scale. Furthermore, the results reveal that social media addiction creates wide-ranging effects on an individual’s psychological, physical, and cognitive processes.

**Conclusion:**

It is concluded that individuals’ social media addiction has a statistically significant impact on their recreation anxiety.

## Introduction

With the rapid advancement of digital technologies, social media platforms have become an integral part of individuals’ daily lives. In a general sense, social media can be defined as internet-based platforms created through the opportunities provided by digital technologies, enabling individuals to communicate and interact with one another (Özdemir, 2019, p. 92). Beyond allowing users to share self-generated content with communities and maintain interaction, these platforms offer opportunities to spend leisure time either actively or passively through activities such as conducting research for information, following current news, playing games for entertainment, watching videos, or shopping (Baş and Diktaş, 2020, p. 197; Erdem and Kutluk Bozkurt, 2023, p. 3512). Numerous social media platforms are popularly used by individuals both globally and in Türkiye (Çiftçi, 2018, p. 418). Among these, the most frequently used platforms in Türkiye are identified as Facebook, Instagram, YouTube, X (formerly Twitter), Pinterest, LinkedIn, and Reddit, respectively (Statcounter Global Stats, 2025).

While social media platforms offer various opportunities and possibilities by overcoming the limitations of time and space, negative conditions such as addiction may also emerge (Çömlekçi and Başol, 2019, p. 174). Addiction can be briefly described as a phenomenon characterized by an individual’s uncontrolled and non-purposeful pursuit of a substance or behavior, increasing the frequency of use over time by developing tolerance, continuing despite negative consequences, and the emergence of withdrawal symptoms during cessation attempts (Uğurlu et al., 2012, p. 38; Yıldız and Koçak, 2020, p. 1105). Social media addiction, on the other hand, is a type of behavioral addiction where an individual utilizes social media to escape the stresses of life; however, despite negative outcomes such as damage to social relationships and withdrawal from activities due to excessive use, the individual remains unable to control this behavior (Uslu, 2021, p. 375). In this context, while social media use can sometimes serve as a tool for socialization, in cases of addiction, it may reflect an asocial tendency as an area of escape from the community (Hazar, 2011, p. 153). To construct a robust theoretical bridge between this behavioral pathology and leisure constraints, these maladaptive digital habits must be mapped directly onto the cognitive and behavioral mechanisms governing non-work behavior. It can be argued that such psychological and social consequences may also affect the ways individuals evaluate their leisure time and their participation processes in recreational activities.

Recreation is defined as active or passive activities in which individuals participate voluntarily during their leisure time to feel well, escape daily routines, and achieve self-actualization (Kurar and Baltacı, 2014, p. 43). It can be stated that individuals participate in recreational activities to rest, maintain health, interact with others, and gain diverse experiences (Er et al., 2019, p. 110). Indeed, these activities are observed to be significant for individuals from cognitive, physiological, and social perspectives (Vurgun, 2019, pp. 19–20). Leisure activities not only support personal development but also contribute to the enhancement of social skills such as cooperation, sharing, and communication by increasing social interactions (Yalçın and Ayhan, 2020, p. 206; Aytan, 2010, p. 3). On the other hand, there are economic, social, and individual barriers that may restrict participation in recreation activities (Orhun et al., 2024, p. 185).

In this framework, in the scale development study titled “recreation anxiety” by Orhun et al. (2024), it was emphasized that factors such as social anxiety, performance anxiety, and recreation constraints should be considered to explain recreation anxiety. They defined the concept of recreation anxiety as the general state of anxiety (feeling worried, tense, and restless) experienced by individuals regarding recreational activities. To provide the conceptual synthesis mandated by multi-dimensional psychological frameworks, this study introduces a theoretical synthesis integrating Social Comparison Theory (Festinger, 1954), Conspicuous Consumption (Veblen, 1899), and the Mean World Syndrome (Gerbner and Gross, 1976). This structured framework systematically explains why virtual platform addiction inherently triggers and exacerbates the six specific sub-dimensions of recreation anxiety. In this context, this study aims to examine the empirical effect of individuals’ social media addiction on recreation anxiety and to contribute a theoretically grounded model to the relevant literature.

## Theoretical framework and hypotheses

### Self-efficacy, social anxiety, and physical symptoms

Within the framework of Social Comparison Theory, individuals possess an innate drive to evaluate their opinions and abilities by comparing themselves with others (Festinger, 1954). In digital spaces, this mechanism often manifests as upward social comparison, where users are incessantly exposed to highly curated, idealized depictions of peers’ leisure experiences. The literature indicates that social media addiction increases levels of depression and social anxiety (Doğan, 2021, p. 20; Aktan, 2018, p. 40) and negatively impacts occupational, academic, and social lives (Güler et al., 2019, p. 2; Demir and Kumcağız, 2019, p. 25). Additionally, conditions such as poor sleep quality, inability to set boundaries, mental preoccupation, and detachment from reality are among the adverse effects of social media addiction on the individual (Kıran et al., 2020, p. 436). An individual spending time in digital environments at an addictive level may also cause social isolation by detaching from reality, subsequently triggering social anxiety (Arslan and Bardakçı, 2021, p. 902). When addicted users evaluate their own abilities against these distorted online standards, their perceived behavioral control diminishes, directly elevating anxiety and lowering self-efficacy during physical leisure execution.

In line with these psychological deformities, literature suggests that increased screen time leads to decreased physical activity, potentially resulting in health issues such as muscle loss, increased fat mass, and obesity (Erdoğanoğlu and Arslan, 2019; Baskan et al., 2023; Khan et al., 2017; Kim et al., 2015). Furthermore, Kuşan (2025) noted that as Body Mass Index (BMI) increases, social media addiction also rises, while cognitive behaviors related to physical activity participation decrease. Consequently, addicted individuals with sedentary lifestyles may manifest physical symptoms such as heart palpitations, fatigue, and tension when considering or engaging in recreational activities. Based on these integrated socio-cognitive and somatic patterns, the following hypotheses are proposed:

*H1:* Individuals’ social media addiction has a statistically significant effect on self-efficacy and social anxiety, which are sub-dimensions of recreational anxiety.

*H2:* Individuals’ social media addiction has a statistically significant effect on physical symptoms, a sub-dimension of recreational anxiety.

### Economic and safety anxieties

Negative emotional states such as depression and anxiety are also factors that limit participation in recreational activities (Orhun et al., 2024, p. 610; Çağlayan and Hassan, 2023, p. 304; Crawford and Godbey, 1987, p. 122). While restricting participation in recreation can lead to social isolation (Orhun et al., 2024, p. 185), specific financial and environmental factors shape these boundaries. The socioeconomic dimension of this interaction can be grounded in the concept of Conspicuous Consumption, which denotes the practice of displaying costly goods and activities to public view to enhance social prestige and secure peer approval (Veblen, 1899). The concept of “Conspicuous Consumption” has transitioned into social media platforms, where individuals showcase luxury vacations, hotels, and recreational activities to enhance status and gain social approval (Bayuk and Öz, 2018). Particularly for individuals with limited or moderate economic resource allocation, this digitally induced perception of luxury leisure is hypothesized to progressively exacerbate relative deprivation and economic anxiety regarding recreational participation.

Concurrently, the environmental dimensions of recreation anxiety can be understood via Cultivation Theory, specifically the Mean World Syndrome, which posits that heavy exposure to unmediated media content cultivates an exaggerated perception of the world as inherently dangerous and untrustworthy (Gerbner and Gross, 1976). This syndrome suggests that frequent exposure to negative media content leads individuals to believe the world is inherently dangerous and people are untrustworthy (Arslan and Çötok, 2023). Altıntaş (2017) found that exposure to violent news online significantly increases anxiety. Thus, social media addicts exposed to constant digital reports of accidents and risks may develop heightened safety concerns regarding recreational participation. Hence, the following hypotheses are developed:

*H3:* Individuals’ social media addiction has a statistically significant effect on economic anxiety, a sub-dimension of recreational anxiety.

*H4:* Individuals’ social media addiction has a statistically significant effect on safety anxiety, a sub-dimension of recreational anxiety.

### Responsibility and anticipatory anxieties

To fully comprehend the structural impact of digital dependency on behavioral paralysis, these findings must be anchored in Self-Determination Theory (SDT; Deci and Ryan, 1985). SDT posits that optimal human functioning and well-being depend on the satisfaction of three basic psychological needs: autonomy, competence, and relatedness. When social media addiction disrupts these needs, individuals often experience a shift from intrinsic motivation to introjected regulation a state where behaviors are driven by internal pressures such as guilt, anxiety, and obligation.

Literature indicates that addicted individuals often struggle with time management and exhibit tendencies toward procrastination regarding their duties (Yılmaz and Korkmaz, 2024; Demirel et al., 2022; Yüksel-Şahin and Altınsoy, 2025). Within the SDT framework, this chronic digital procrastination compromises the individual’s sense of autonomy and competence. Consequently, recreation is perceived not as an autonomous, restorative, or motivating activity, but rather as an unearned indulgence that generates intrapsychic guilt stemming from neglected real-world responsibilities. This motivational distortion shifts the core meaning of leisure from active rejuvenation to an anxiety-inducing reminder of undone operational tasks, thereby triggering intense responsibility anxiety. Furthermore, this disruption extends to the temporal and cognitive evaluation of leisure outcomes, which can be grounded in the concept of Delay Discounting (Ainslie, 2001) within motivational psychology. Social media platforms fundamentally alter cognitive reward processing by providing instant gratification, such as likes and comments, which triggers rapid dopamine release (Çağlayan and Arslantaş, 2023; Emre, 2024; Aksu and Karaca Bıçakçı, 2025). Dopamine regulates motivation and the subjective sense of reward (Güzel et al., 2019; Tiring and Tiring, 2025). Bypassing natural reward thresholds through perpetual digital reinforcement accelerates delay discounting; that is, individuals devalue long-term, non-instantaneous rewards in favor of immediate ones.

When individuals accustomed to this rapid digital reward system engage in recreational activities which inherently require temporal investment, physical effort, or delayed psychological outcomes they develop unrealistic expectations for immediate and tangible benefits. When these results are not instantaneous, a cognitive dissonance occurs, directly leading to anticipatory anxiety regarding the efficacy of their leisure behavior. Consequently, the following final hypotheses are established:

*H5:* Individuals’ social media addiction has a statistically significant effect on responsibility anxiety, a sub-dimension of recreational anxiety.

*H6:* Individuals’ social media addiction has a statistically significant effect on anticipatory anxiety, a sub-dimension of recreational anxiety.

## Method

### Research model

**Figure 1:**
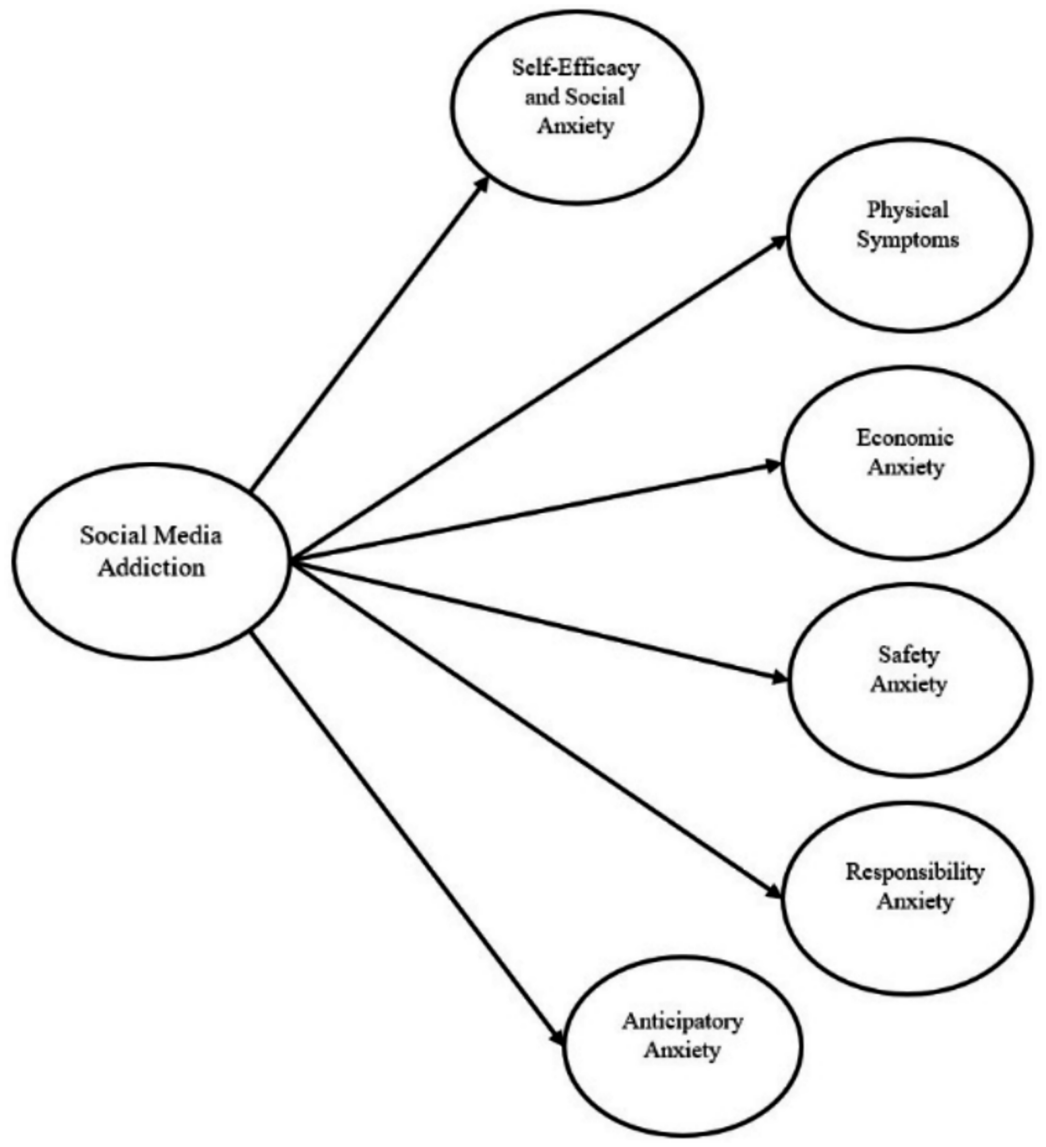
Research model.

In this study, the descriptive survey model, one of the quantitative research methods, was utilized to identify and examine the impact of individuals’ social media addiction on recreation anxiety. This model is a research approach in which a phenomenon that occurred in the past or continues to exist is described as it is, without any intervention or modification (Karasar, 2012). Accordingly, the study sought an answer to the research question: “Does individuals’ social media addiction have an effect on recreation anxiety?” Based on this primary question, the following hypotheses were formulated (Figure 1):

*H1:* Individuals’ social media addiction has a statistically significant effect on self-efficacy and social anxiety, which are sub-dimensions of recreational anxiety.

*H2:* Individuals’ social media addiction has a statistically significant effect on physical symptoms, a sub-dimension of recreational anxiety.

*H3:* Individuals’ social media addiction has a statistically significant effect on economic anxiety, a sub-dimension of recreational anxiety.

*H4:* Individuals’ social media addiction has a statistically significant effect on safety anxiety, a sub-dimension of recreational anxiety.

*H5:* Individuals’ social media addiction has a statistically significant effect on responsibility anxiety, a sub-dimension of recreational anxiety.

*H6:* Individuals’ social media addiction has a statistically significant effect on anticipatory anxiety, a sub-dimension of recreational anxiety.

### Research population and sample

The target population of this study comprises adult individuals residing in the province of Çanakkale, Türkiye. Due to the lack of an exhaustive sampling frame encompassing the entire regional population, a non-probability convenience sampling method was utilized to recruit participants based on accessibility and voluntary cooperation. A total of 399 volunteer adult individuals (160 males, 40.1%; 239 females, 59.9%) participated in the research. To evaluate the statistical adequacy and power of the sample size, a post-hoc verification was conducted based on population representation formulas. According to Yazıcıoğlu and Erdoğan (2004), for a large or infinite population, a minimum sample size of 384 participants is statistically sufficient to represent the population within a 95% confidence interval and a 5% margin of error (*p* = 0.50, *q* = 0.50, *d* = 0.05). Since the final sample size (*N* = 399) exceeds this conventional threshold, it can be empirically stated that the sample provides adequate statistical power and satisfactory representation for the structural equation modeling (SEM) analyses, thereby minimizing potential sampling bias.

### Data collection tools

*Personal information form*: A Personal Information Form was utilized to collect data regarding the participants’ age, gender, income level, duration of recreational social media use, and frequency of participation in recreational activities.

*Social media addiction scale*: To examine social media addiction, the “Social Media Addiction Scale,” developed by Çömlekçi and Başol (2019), was employed. The scale consists of a total of 7 items and is designed in a 5-point Likert-type format (1: Never - 5: Always). The scale has a unidimensional (single-factor) structure.

*Recreation anxiety scale*: The “Recreation Anxiety Scale,” developed by Orhun et al. (2024), was used to measure individuals’ recreation anxiety. The scale comprises 25 items and follows a 5-point Likert-type format (1: Strongly disagree - 5: Strongly agree). It consists of six sub-dimensions:

- Self-efficacy and Social Anxiety (Items 1–7),
- Physical Symptoms (Items 8–11),
- Economic Anxiety (Items 12–15),
- Safety Anxiety (Items 16–18),
- Responsibility Anxiety (Items 19–22),
- Anticipatory Anxiety (Items 23–25).

There are no reverse-coded items in the scale.

### Common method bias

Since the empirical data for both independent and dependent variables were collected from the same self-reported source during a single cross-sectional assessment, the potential risk of common method bias (CMB) was methodologically evaluated. Following the statistical recommendations outlined by Podsakoff et al. (2003), Harman’s (1976) Single-Factor Test was performed via exploratory factor analysis (EFA). All 32 items representing the latent constructs were forced into an unrotated, single-factor solution. The statistical extraction revealed that no single dominant factor accounted for the majority of the variance, with the first principal factor explaining only 41.95% of the total variance. Because this value falls substantially below the conservative threshold of 50.0%, it can be empirically verified that common method bias does not pose a substantial threat to the validity of the structural relationships evaluated in this research, thereby confirming the robustness of the structural equation modeling (SEM) estimations.

### Data analysis

Various statistical methods were employed in the analysis of the data obtained in the study. The data transferred to SPSS were initially screened for missing values and data entry errors; no corrections were found to be necessary. Subsequently, before proceeding to hypothesis testing, fundamental assumptions specifically the normality of the data were examined, with skewness and kurtosis values being evaluated within this scope. Following the confirmation of normality, validity and reliability analyses of the measurement model were conducted. For reliability, Cronbach’s Alpha and Composite Reliability (CR) values were utilized. For validity, convergent and discriminant validity criteria were applied. Convergent validity was tested through AVE (Average Variance Extracted) and CR results, while discriminant validity was assessed by comparing AVE and MSV (Maximum Shared Variance) values. Furthermore, construct validity was evaluated using Confirmatory Factor Analysis (CFA), employing fit indices such as Chi-square (*χ*^2^), NFI, GFI, CFI, RMSEA, and SRMR. In the final stage, Structural Equation Modeling (SEM) was applied to test the theoretical model. In this analysis, performed using the AMOS software, the effects of exogenous variables on endogenous variables were evaluated simultaneously, and the overall model fit was tested.

## Results

Upon examining the demographic and behavioral profiles of the 399 participants, it was determined that 239 (59.9%) were female and 160 (40.1%) were male. Regarding the age distribution, 70 individuals (17.5%) were in the 18–20 age group, 89 (22.3%) were in the 21–24 age group, and the majority, 240 participants (60.2%), were aged 25 and above. In terms of income status, 59 participants (14.8%) were in the low-income group, 285 (71.4%) were in the middle-income group, and 55 (13.8%) were in the high-income group (Table 1).

**Table 1:** Descriptive statistics.

Variables	Groups	n	%
Gender	Female	239	59.9
	Male	160	40.1
Age	18–20 years	70	17.5
	21–24 years	89	22.3
	25 years and above	240	60.2
Income level	Low	59	14.8
	Medium	285	71.4
	High	55	13.8
Average daily social media usage	1–3 h	160	40.1
	3–6 h	173	43.4
	7 h and above	66	16.5
Frequency of participation in recreational activities	No participation in recreational activities	153	38.3
	1 day per week	105	26.3
	2–3 days per week	99	24.8
	4–5 days per week	33	8.3
	6–7 days per week	9	2.3
Total		399	100

Analysis of daily social media usage duration revealed that 160 individuals (40.1%) spent 1–3 h, 173 (43.4%) spent 3–6 h, and 66 (16.5%) spent 7 h or more on social media platforms. Regarding the frequency of participation in recreational activities, 153 participants (38.3%) reported no participation in any activities, while 105 (26.3%) participated 1 day per week, 99 (24.8%) 2–3 days per week, 33 (8.3%) 4–5 days per week, and 9 (2.3%) 6–7 days per week.

Upon examining the normality, reliability, and validity results presented in Table 2, it was determined that univariate normality was ensured, as the skewness and kurtosis values remained within the acceptable thresholds of ±1.5. In terms of reliability, the finding that Cronbach’s Alpha (*α*) and Composite Reliability (CR) values for all variables exceeded 0.70 indicates that the scales possess high internal consistency; this result is further corroborated by the MaxR(H) values. In the validity analysis, Average Variance Extracted (AVE) values above 0.50 confirmed that convergent validity was achieved. Furthermore, the fact that Maximum Shared Variance (MSV) values were generally lower than the corresponding AVE values demonstrates that discriminant validity was largely established. However, the close proximity of the AVE (0.518) and MSV (0.507) values for the “responsibility” dimension suggests a relative weakness in the discriminant validity of this specific sub-dimension. Overall, the table indicates that the measurement instrument exhibits high reliability, satisfactory validity, and appropriate normality characteristics; consequently, it constitutes a statistically favorable tool for advanced structural equation modeling analyses.

**Table 2:** Normality, reliability, and validity analyses.

Variables	*f*	*α*	CR	AVE	MSV	MaxR(H)	Skewness	Kurtosis
Social media addiction	7	0.941	0.941	0.695	0.334	0.948	0.436	−1.188
Self-efficacy and social anxiety	7	0.913	0.915	0.607	0.507	0.922	0.608	−0.396
Physical symptoms	4	0.932	0.933	0.777	0.450	0.937	0.858	−0.061
Economic anxiety	4	0.897	0.899	0.692	0.507	0.912	0.342	−0.655
Safety anxiety	3	0.919	0.919	0.791	0.479	0.920	0.538	−0.639
Responsibility anxiety	4	0.846	0.811	0.518	0.507	0.812	0.526	−0.470
Anticipatory anxiety	3	0.849	0.853	0.660	0.394	0.860	0.077	−0.801

*α*: Cronbach’s Alpha coefficient (internal consistency), CR: composite reliability, AVE: average variance extracted, MSV: maximum shared variance, MaxR(H): maximal reliability coefficient.

Table 3 presents the discriminant validity of the variables based on the Fornell–Larcker criterion. While the diagonal values represent the square root of the Average Variance Extracted (AVE) for each variable, the correlation coefficients below the diagonal indicate the inter-construct relationships. According to Fornell and Larcker (1981), discriminant validity is established if the square root of the AVE of a construct is greater than its correlations with other constructs. Upon examination of the table, it is observed that the square root of the AVE for each variable (e.g., 0.833 for social media addiction; 0.779 for self-efficacy/social anxiety; 0.882 for physical symptoms; 0.832 for economic anxiety; 0.889 for safety anxiety; 0.720 for responsibility anxiety; and 0.812 for anticipatory anxiety) is higher than the correlation values in its respective row and column. This indicates that, although there are statistically significant relationships between the constructs (*p* < 0.001), each construct is conceptually distinct within the measurement model, thereby representing a unique concept.

**Table 3:** Discriminant validity results.

Variables	1	2	3	4	5	6	7
1. Social media addiction	0.833*	.	.	.	.	.	.
2. Self-efficacy and social anxiety	0.512***	0.779*	.	.	.	.	.
3. Physical symptoms	0.467***	0.671***	0.882*	.	.	.	.
4. Economic anxiety	0.481***	0.507***	0.448***	0.832*	.	.	.
5. Safety anxiety	0.450***	0.612***	0.568***	0.517***	0.889*	.	.
6. Responsibility anxiety	0.578***	0.712***	0.638***	0.712***	0.692***	0.720*	.
7. Anticipatory anxiety	0.241***	0.433***	0.339***	0.502***	0.434***	0.627***	0.812*

*Fornell ve Larcker. The italicized values on the diagonal represent the square root of the average variance extracted (AVE) according to the Fornell-Larcker criterion.

**Figure 2:**
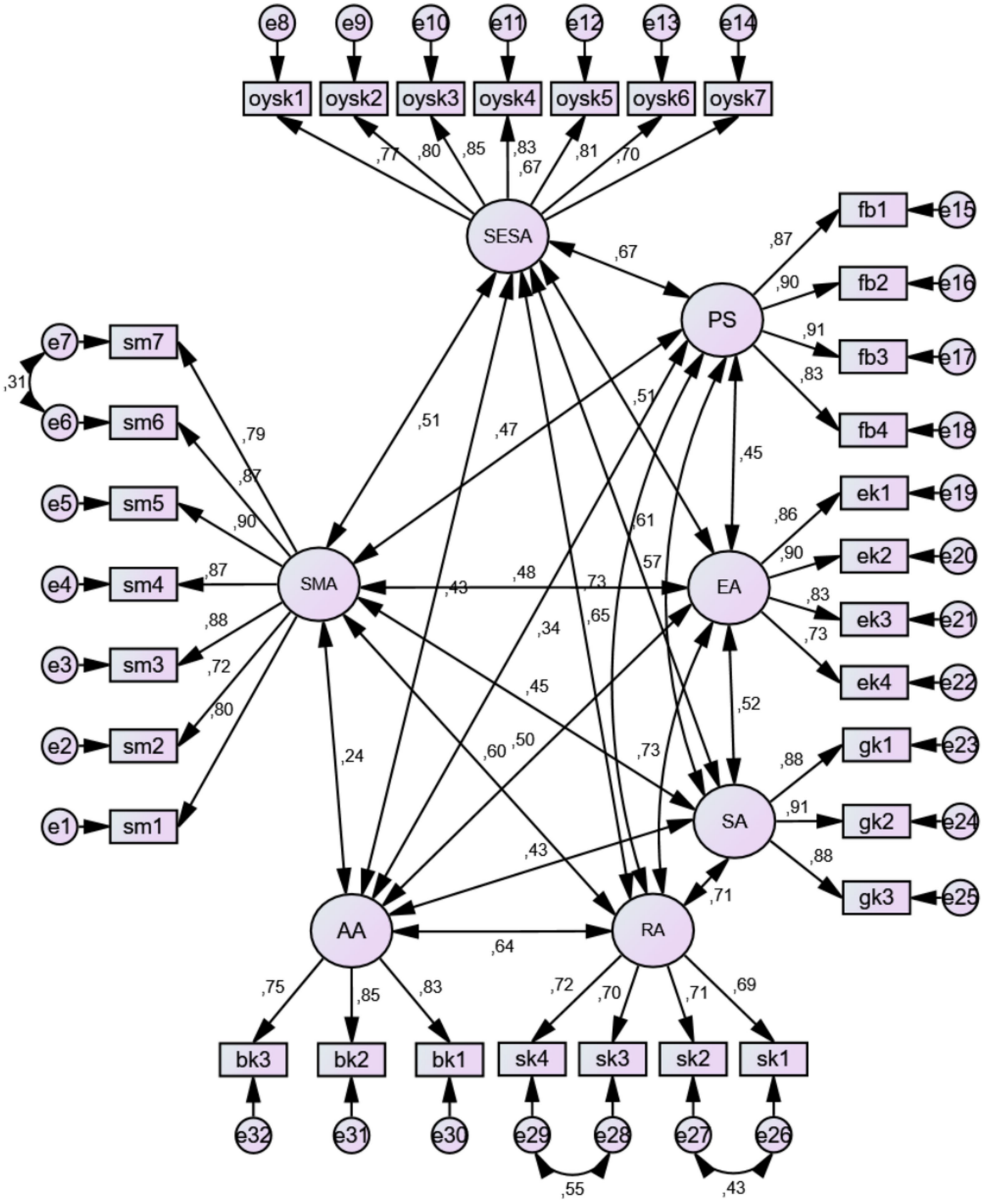
Path diagram for the confirmatory factor analysis (CFA).

The Confirmatory Factor Analysis (CFA) model fit diagram presented in Figure 2 illustrates the construct validity of the measurement model employed in the study and the statistical consistency of the relationships between variables. As shown in the diagram, each latent variable (e.g., social media addiction, self-efficacy/anxiety, physical, economic, safety, responsibility, and anticipatory anxiety) is strongly associated with its respective observed variables (indicators). The vast majority of factor loadings are above 0.70, with several ranging between 0.80 and 0.90, indicating that the measurement model possesses a high degree of convergent validity. The covariance paths between the latent variables are positive and significant, demonstrating a pattern of constructs that are conceptually related yet distinct. Furthermore, the low error terms observed in the model and the stability of the standardized factor loadings suggest that measurement error is minimal and the model exhibits a good statistical fit.

Table 4 presents the model fit indices regarding the Confirmatory Factor Analysis (CFA). The results obtained indicate that the model exhibits a good level of fit with the data. First, the CMIN/DF (*χ*^2^/df) ratio of 2.020 demonstrates that the model has an acceptable level of fit, as it satisfies the recommended threshold of being below 3. Although the GFI (0.881) value is slightly below the 0.90 threshold, it exhibits a marginal yet acceptable level of fit.

**Table 4:** Model fit indices of the measurement model.

CMIN/DF	*p*	DF	GFI	NFI	CFI	RMSEA	SRMR
2.020	0.000	440	0.881	0.916	0.955	0.051	0.041

*p* < 0.05; GFI, NFI, CFI > 0.900; RMSEA < 0.08; SRMR < 0.08.

Conversely, the NFI (0.916) and CFI (0.955) values are above 0.90, indicating that the model is robust in terms of comparative fit. Furthermore, the fact that the RMSEA (0.051) and SRMR (0.041) values are below the 0.08 limit demonstrates a low error level and high fit quality. Overall, these indicators reveal that the CFA model exhibits a satisfactory and statistically robust fit; therefore, the measurement model represents a valid, reliable, and theoretically supported structure.

**Figure 3:**
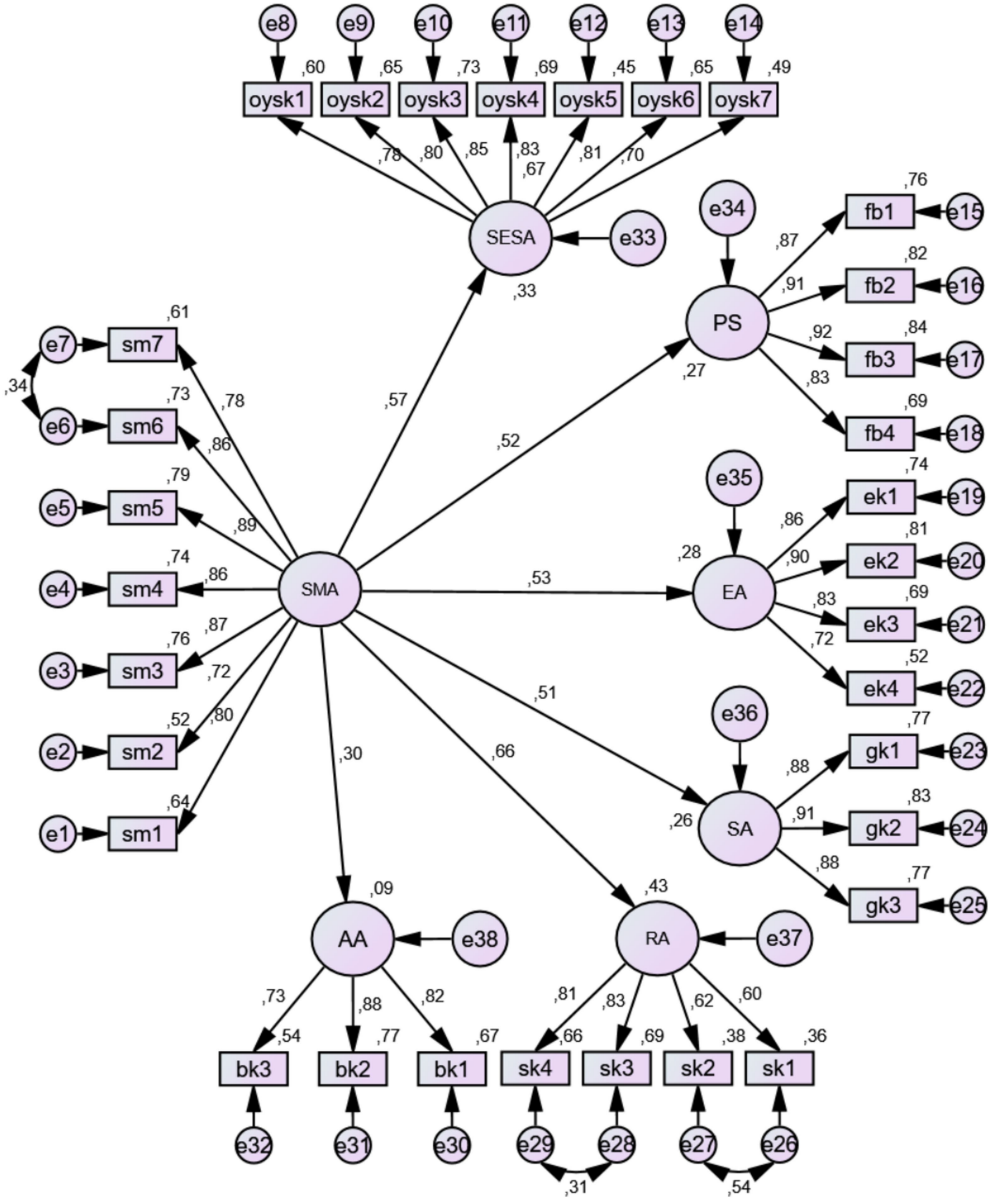
Results of the structural equation model (SEM).

The Structural Equation Modeling (SEM) diagram presented in Figure 3 illustrates the causal relationships and the statistical power of the theoretical model tested in the research. In the model, the “social media addiction” variable is observed to exert direct and indirect effects on the other latent variables. Specifically, social media use has positive standardized effects of 0.57 on “self-efficacy/social anxiety,” 0.52 on “physical symptoms,” 0.53 on “economic anxiety,” 0.51 on “safety anxiety,” 0.66 on “responsibility anxiety,” and 0.30 on “anticipatory anxiety.” These values indicate that the social media variable assumes a central predictor role within the model. The fact that all path coefficients are positive and statistically significant suggests that the hypothesized relationships in the model are empirically supported.

According to the structural equation modeling (SEM) results presented in Table 5, the social media addiction variable significantly and positively influences all sub-dimensions within the model (*p* < 0.001). Based on the obtained standardized *β* coefficients, the strongest effect of social media addiction was observed on responsibility anxiety (*β* = 0.659), while its weakest effect was on anticipatory anxiety (*β* = 0.299). When examining the explained variance (*R*^2^) values, it is evident that the model accounts for approximately 43% of the variance in responsibility anxiety and 32% of the variance in self-efficacy and social anxiety. Consequently, all hypotheses proposed within the structural model were empirically supported.

**Table 5:** Results of hypothesis testing for the structural model.

Path/Relationship	*β*	*B*	S.H.	K.O.	*p*	*R* ^2^	Hypothesis/Result
Social Media Addiction ➔ Self-efficacy and Social Anxiety	0.572	0.470	0.045	10,371	***	0.327	H1: supported
Social Media Addiction ➔ Physical Symptoms	0.524	0.508	0.051	10,046	***	0.275	H2: supported
Social Media Addiction ➔ Economic Anxiety	0.533	0.534	0.053	10,049	***	0.285	H3: supported
Social Media Addiction ➔ Safety Anxiety	0.512	0.518	0.053	9,736	***	0.262	H4: supported
Social Media Addiction ➔ Responsibility Anxiety	0.659	0.454	0.055	8,267	***	0.434	H5: supported
Social Media Addiction ➔ Anticipatory Anxiety	0.299	0.293	0.054	5,379	***	0.089	H6: supported

*β*: standardized beta coefficient; *B*: unstandardized coefficient; S.E.: standard error; C.R.: critical ratio (*t*-value); ***p < 0.001.

## Discussion and conclusion

The primary objective of this study was to determine the impact of social media addiction on recreation anxiety. The participant profile predominantly consisted of middle-income females aged 25 and above, with daily social media usage concentrated between 3 and 6 h and participation in recreational activities generally limited to 0–1 days per week. The analysis of data obtained from this non-probability convenience sample yielded several significant findings. All hypotheses tested within the framework of Structural Equation Modeling (SEM) were found to be significant (*p* < 0.001). As illustrated in Table 5, social media addiction exerted its strongest effect on responsibility anxiety, suggesting that individuals experience an increase in feelings of responsibility or responsibility-related anxieties due to their social media use. Other significant effects were observed, in descending order, on self-efficacy and social anxiety, economic anxiety, physical symptoms, and safety anxiety, while the weakest effect was found on anticipatory anxiety. These findings reveal that social media addiction has wide-ranging impacts on individuals’ psychological, physical, and cognitive dimensions, demonstrating the overall robust explanatory power of the model. Regarding the first hypothesis (H1), it was determined that social media addiction has a statistically significant effect on the self-efficacy and social anxiety sub-dimension. These finding parallels existing literature. Kandasamy et al. (2019) identified a significant relationship between internet addiction and anxiety in a study of 200 students (mean age 18.5), finding that students with high levels of addiction experienced anxiety, fear, and tension. Similarly, Weinstein et al. (2015) found a significant correlation between internet addiction and social anxiety among 120 university students, where social anxiety increased alongside addiction levels. Ağırtaş and Güler (2020) reported that students who preferred social media over spending time with friends exhibited higher social anxiety, identifying a moderate positive correlation between internet addiction and social anxiety. From the perspective of Social Comparison Theory (Festinger, 1954), individuals who evaluate themselves against others perceived as “superior” on social media may experience diminished self-efficacy and heightened social anxiety (Büyükmumcu and Ceyhan, 2019). In line with the second hypothesis (H2), social media addiction was found to significantly affect physical symptoms. Literature suggests that increased screen time leads to decreased physical activity, potentially resulting in health issues such as muscle loss, increased fat mass, and obesity (Erdoğanoğlu and Arslan, 2019; Baskan et al., 2023; Khan et al., 2017; Kim et al., 2015). Furthermore, Kuşan (2025) noted that as Body Mass Index (BMI) increases, social media addiction also rises, while cognitive behaviors related to physical activity participation decrease. Consequently, addicted individuals with sedentary lifestyles may manifest physical symptoms such as heart palpitations, fatigue, and tension when considering or engaging in recreational activities. The findings for the third hypothesis (H3) indicate that social media addiction significantly impacts economic anxiety. The concept of “Conspicuous Consumption” (Veblen, 1899) has transitioned into social media platforms, where individuals showcase luxury vacations, hotels, and recreational activities to enhance status and gain social approval (Bayuk and Öz, 2018). Such conspicuous displays can create a perception that recreational activities are inherently costly. Given that 71.4% of the participants in this study were middle-income individuals, this perception likely exacerbates economic anxiety regarding recreation. The fourth hypothesis (H4) revealed a significant effect on safety anxiety, which can be explained by Gerbner’s “Mean World Syndrome.” This syndrome suggests that frequent exposure to negative media content leads individuals to believe the world is inherently dangerous and people are untrustworthy (Arslan and Çötok, 2023). Altıntaş (2017) found that exposure to violent news online significantly increases anxiety. Thus, social media addicts exposed to constant digital reports of accidents and risks may develop heightened safety concerns regarding recreational participation. Regarding the fifth hypothesis (H5), social media addiction significantly influenced responsibility anxiety. Literature indicates that addicted individuals often struggle with time management and exhibit tendencies toward procrastination regarding their duties (Yılmaz and Korkmaz, 2024; Demirel et al., 2022; Yüksel-Şahin and Altınsoy, 2025). In this context, recreation may be perceived not as a restorative or motivating activity, but as a source of guilt stemming from neglected responsibilities, thereby triggering responsibility anxiety. Finally, for the sixth hypothesis (H6), a significant effect was found on anticipatory anxiety. Social media platforms trigger the brain’s reward system by providing instant gratification such as likes and comments and dopamine release (Çağlayan and Arslantaş, 2023; Emre, 2024; Aksu and Karaca Bıçakçı, 2025). Dopamine regulates motivation and the sense of reward (Güzel et al., 2019; Tiring and Tiring, 2025). Individuals accustomed to this rapid digital reward system may develop unrealistic expectations for immediate and tangible benefits from recreational activities, leading to anticipatory anxiety when such results are not instantaneous. In conclusion, social media addiction serves as a significant predictor of various dimensions of recreation anxiety.

### Theoretical implications

The empirical findings of this study offer substantial theoretical contributions to recreational behavior psychology by establishing an innovative conceptual bridge between digital addiction literature and leisure constraints frameworks. The structural equation modeling (SEM) estimations demonstrate that social media addiction does not merely act as a superficial time deficit; rather, it paralyzes individuals’ leisure experiences through intricate psychological and cognitive mechanisms. The detection of the highest path coefficient on responsibility anxiety provides a critical theoretical expansion within the context of Self-Determination Theory (Deci and Ryan, 1985). Social media dependency sabotages baseline psychological needs namely autonomy and competence shifting leisure motivation from intrinsic drives to introjected regulation. Addicted individuals exhibiting chronic digital procrastination perceive recreational activities not as spaces for autonomous rejuvenation, but as sources of intense intrapsychic guilt stemming from neglected real-world operational duties (academic, professional, or domestic tasks). Consequently, leisure is stripped of its emancipatory essence and transformed into an emotional burden. Concurrently, the weakest yet statistically significant effect on anticipatory anxiety can be robustly grounded in Delay Discounting theory (Ainslie, 2001). The micro-dopaminergic instant gratification loops embedded in social platforms disrupt the human reward-processing system. Recreational activities, which inherently necessitate temporal investment, physical exertion, and delayed psychological outcomes, are cognitively devalued by brains accustomed to immediate digital reinforcement. This maladaptive processing generates a cognitive dissonance manifested as anticipatory anxiety regarding the efficacy of physical leisure. Finally, the validation of economic and safety anxieties via Conspicuous Consumption (Veblen, 1899) and the Mean World Syndrome (Gerbner and Gross, 1976) empirically demonstrates how virtual perceptual distortions materialize into concrete, structural barriers within real-world leisure execution.

### Practical implications

Based on the empirical findings, the following practical recommendations are proposed:

- Given that 38.3% of the participants never engage in recreational activities and social media triggers economic anxiety, local authorities are recommended to increase the availability and promotion of existing free public events (such as festivals and nature walks). This approach effectively helps deconstruct the online perception that leisure activities are inherently expensive.
- Since responsibility anxiety represents the primary behavioral barrier, incorporating “Digital Detox” elements or phone-free challenges into current recreation programs could be highly beneficial. This integration helps users overcome subjective time-management pressures and disconnect from screens.

### Limitations and future directions

Despite its contributions, this study possesses several limitations that open avenues for future research. First, the cross-sectional nature of the data restricts the ability to establish definitive causal relationships over time; therefore, future studies could adopt longitudinal frameworks to track the shifting dynamics between digital habits and recreation anxiety. Second, data collection was limited to adult individuals residing in the province of Çanakkale, Türkiye, using a non-probability convenience sampling method. Future academic endeavors should focus on recruiting larger, more diverse, and stratified probability samples across different regions to enhance the generalizability of the findings. Finally, while this research focused heavily on psychological, safety, and economic anxieties, upcoming studies could investigate the mitigating effects of specific intervention strategies, such as structured digital detox programs, on clinical populations.

## Data Availability

The dataset generated and/or analyzed during the current study is not publicly available due to certain restrictions, but is available from the corresponding author on reasonable request. Requests to access these datasets should be directed to ramazanozavciii@gmail.com.
